# DLLT: A dual-layer LSTM-transformer model for real-time energy and dynamics prediction in plug-in hybrid electric vehicles

**DOI:** 10.1371/journal.pone.0335542

**Published:** 2025-11-05

**Authors:** Xuezhao Zhang, Zijie Chen, Xiaofen Fang

**Affiliations:** 1 Faculty of Mechanical and Electrical Engineering, Quzhou College of Technology, Quzhou, Zhejiang, China; 2 School of Computer Science and Engineering, Faculty of Innovation Engineering, Macau University of Science and Technology, Macau, China; National University of Singapore, SINGAPORE

## Abstract

Plug-in Hybrid Electric Vehicles (PHEVs) are increasingly favored for their low emissions and freedom from range anxiety, combining electric efficiency with the extended range of a gasoline engine. While previous research on PHEV energy consumption has predominantly focused on powertrain design and energy management strategies, there is growing recognition of the critical role played by driver behavior in determining real-world energy efficiency. Based on multi-mode vehicle data collected from real-world driving scenarios, we propose a novel dual-layer LSTM-Transformer model, named DLLT, for real-time prediction of energy consumption and driving dynamics in multi-mode PHEVs. The first layer employs an LSTM network to perform mode clustering, while the second layer conducts energy consumption regression using a Transformer model with integrated mode information. This hierarchical architecture enables adaptation to diverse driving and braking modes, significantly enhancing the model’s ability to accurately identify vehicle operation modes and precisely predict energy consumption. To more accurately validate the effectiveness of DLLT in modeling eco-driving behavior for PHEVs, we collected a large amount of multidimensional time-series data from real-world PHEVs. Experimental results demonstrate that the model achieves a 93% accuracy rate in vehicle mode prediction. Under unseen driving conditions, it attains *R*^2^ values of 0.99 for fuel consumption, 0.86 for acceleration, and 0.81 for electric power, outperforming existing models across all evaluation metrics. With its high prediction accuracy and robust generalization capability, DLLT shows great potential for applications in PHEV eco-driving behavior analysis, intelligent energy management systems, and future autonomous driving control strategies.

## 1 Introduction

Energy conservation and emission reduction have become central objectives in the global automotive industry, where vehicle intelligence plays a pivotal role in advancing sustainability [1,2]. Road vehicles accounts for a significant portion of total greenhouse gas emissions [3,4]. In existing transportation systems, enhancing driver behavior and promoting energy-efficient driving practices are among the most effective strategies to achieve these environmental goals. In parallel, the automotive industry is shifting toward the development of new energy vehicles (NEVs), such as battery electric vehicles (EVs) and PHEVs, to address climate challenges and support carbon neutrality [5–8]. Studies indicate that autonomous vehicles equipped with advanced driving automation systems can reduce greenhouse gas emissions and pollutants by at least 20% compared to human-driven vehicles [9,10]. Whether aiming to improve existing drivers’ behaviors or to deploy autonomous systems in next-generation NEVs, it is essential to model the intricate relationship between driver actions and vehicle energy consumption. This highlights the necessity of developing vehicle models grounded in real-world driver behavior data.

**Fig 1:**
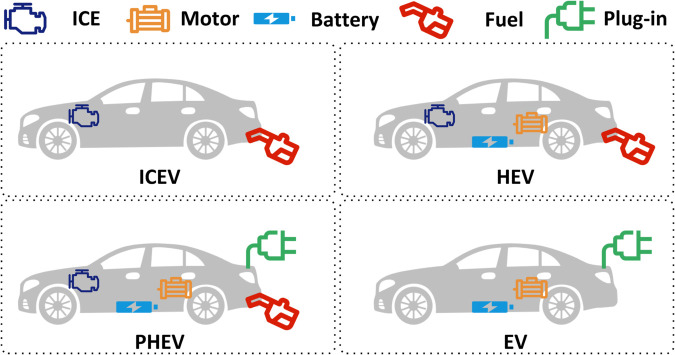
Comparison of vehicle powertrain types: ICEV, HEV, PHEV, and EV.

The mainstream vehicles currently available in the market can be broadly classified according to their powertrain configurations into four categories: internal combustion engine vehicles (ICEVs), hybrid electric vehicles (HEVs), PHEVs, and EVs. A comparison of these four powertrain types is shown in Fig 1. For all configurations, optimized energy management strategies are commonly adopted to improve energy efficiency and reduce emissions. The optimization process typically involves two key steps: first, establishing a white-box model of the vehicle and its powertrain system; and second, designing the energy management strategy using optimization algorithms. Extensive research has been done on white-box modeling. Engine models range from detailed combustion simulations [11,12] to simpler map-based empirical ones [13]. Transmissions are often modeled using efficiency losses from MAP data [14]. Motors can be modeled with physics-based equations or power-loss maps [15]. Battery models focus on electrochemistry, aging, and thermal effects [16]. For whole-vehicle energy modeling, two methods are common. Backward simulation calculates energy needs from resistance forces, skipping driver inputs [17]. Forward simulation includes driver behavior to simulate real-world conditions and trace power demands through the system [18]. These models support the evaluation and tuning of energy management strategies like ECMS or reinforcement learning [19], aiming to minimize energy use under real driving conditions.

Optimizing energy management strategies is crucial for better efficiency. However, its potential for major energy savings is limited if applied only after the vehicle is designed. Therefore, improving how drivers behave remains a key way to save energy and reduce emissions. Recent studies have employed virtual driving simulators to collect driver behavior data under controlled conditions, leveraging deep learning techniques to identify eco-driving patterns. These methods have effectively distinguished between fuel-efficient and high-consumption driving styles in ICEVs [20]. However, their performance tends to degrade when applied to PHEVs. Unlike ICEVs, PHEVs involve multiple power sources and complex energy distribution dynamics, making traditional modeling approaches inadequate for capturing the intricate interactions between driving behavior and energy consumption [21]. Moreover, most existing methods are designed for post-hoc analysis and lack the capability to provide real-time feedback or guidance to drivers. An alternative approach transforms driving behavior from the time domain to the frequency domain, classifying driving styles based on the spectral characteristics of speed fluctuations [22]. Although this method has been applied in PHEV-related research, it often fails to capture detailed electric energy consumption information, with fuel consumption estimates typically being approximations, leading to incomplete or less reliable energy assessments. For EVs, recent efforts have integrated LSTM networks with Transformer architectures to develop comprehensive energy consumption prediction models that consider vehicle status, environmental conditions, and driving behavior. Nevertheless, these models are constrained by low sampling frequencies (e.g., 0.05 Hz) and data segmentation practices that disrupt temporal continuity, thereby limiting their predictive accuracy. Additionally, due to the relatively simple powertrain configurations of EVs, such models struggle to capture the complex nonlinear interactions and multi-source power coupling inherent in PHEVs [23,24]. Some studies have explored dual-layer LSTM architectures for modeling PHEV energy consumption, aiming to better capture temporal dependencies in driving behavior. However, these models neglect critical factors such as battery state-of-charge (SOC) dynamics and the intricate mechanisms of power allocation among multiple energy sources, ultimately limiting their predictive performance and practical applicability [25].

In summary, there are several key limitations to existing driver behavior-based models for predicting vehicle energy consumption and dynamics. A significant limitation is the inability to provide time-step-level predictions, which restricts their applicability for real-time eco-driving feedback. Furthermore, critical variables such as instantaneous fuel consumption and battery power are frequently estimated as opposed to being measured directly, a practice that has the potential to result in inaccuracies. Furthermore, a considerable number of models fail to take into account the temporal dependencies associated with SOC, which has a substantial impact on energy management strategies in PHEVs. Finally, extant approaches have a tendency to underrepresent the complex power distribution dynamics among multiple energy sources, thereby failing to adequately capture the nuanced effects of multi-mode driving on energy allocation decisions.

Therefore, the aim of this paper is to develop a novel energy and dynamics prediction model for PHEVs, based on real-world data covering driver behaviours, vehicle states and energy consumption patterns. The proposed model is intended to deliver highly accurate predictions of both vehicle acceleration and energy consumption indicators. Furthermore, it is designed to effectively capture the temporal dependencies inherent in electric energy consumption during PHEV operations and to faithfully simulate the intricate power distribution dynamics across multiple energy sources. Specifically, we propose a dual-layer learning model called DLLT that combines LSTM and Transformer networks. The architecture follows a two-stage structure: the first layer employs an LSTM network to classify vehicle operating modes, while the second layer incorporates the resulting mode features into a Transformer-based regression framework to perform high-precision prediction. Through this hierarchical modeling strategy and integrated feature fusion mechanism, DLLT can effectively predict energy and dynamics across various operational modes in PHEVs. This paper contributes not only a novel methodological framework for energy and dynamics prediction during the operational phase of PHEVs, but also establishes a theoretical and technical foundation for data-driven energy management strategies and eco-driving control systems in future autonomous and connected vehicles. The primary contributions of this work are as follows.

1. We propose a driver behavior-based model to predict PHEV energy and dynamics using real-world vehicle data. This approach reduces reliance on comprehensive physical parameters required by traditional models, offering a novel way to analyze energy and dynamics during vehicle operation.
2. We propose a dual-layer LSTM–Transformer model, named DLLT, that synergistically combines LSTM and Transformer networks. The LSTM layer identifies vehicle operating modes. The Transformer layer fuses these mode features with driver behavior signals to enable highly accurate multi-output predictions, including vehicle acceleration, fuel consumption, and battery power.
3. DLLT captures the complex nonlinear characteristics of PHEV energy consumption, explicitly modeling the temporal effects of battery SOC variations. This enables a realistic reconstruction of energy flow dynamics under multi-source powertrain coupling, enhancing the fidelity of energy consumption estimation.
4. Extensive experimental evaluations confirm that DLLT significantly outperforms existing state-of-the-art models across multiple performance indicators. The model achieves an *R*^2^ score of 0.99 for fuel consumption prediction, 0.86 for acceleration, and 0.81 for battery power, demonstrating both high predictive accuracy and strong generalization capabilities.

The organization of this paper is as follows: The introduction provides a comprehensive review of the current state of research on eco-driving behavior modeling. Sect 2 presents a detailed analysis of the challenges and issues in PHEVs models based on driver behavior. Sect 3 introduces the design concept and technical approach for the PHEV model training architecture. Sect 4 describes the model training process and offers an in-depth analysis of the experimental results. Sect 5 presents a comprehensive discussion of the model’s strengths, weaknesses, opportunities, and threats. Finally, Sect 6 concludes the paper with a summary of the main findings.

## 2 Driver behavior-based PHEV model

**Fig 2:**
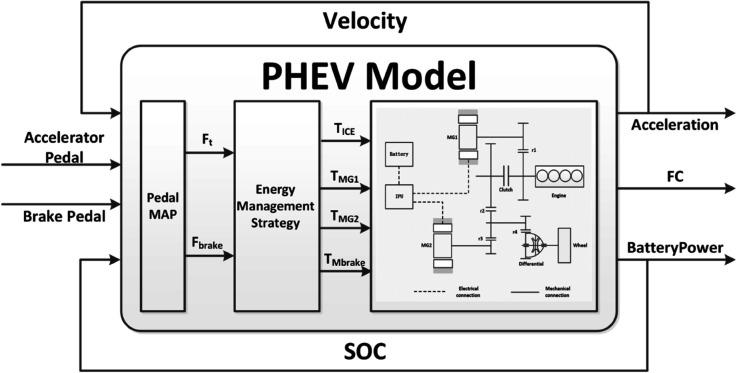
An overview of the driving behavior-based PHEV model architecture.

A PHEV model based on driver behavior is developed through a structured multi-stage process, as illustrated in Fig 2. Driver behavior is primarily represented by two input signals: accelerator pedal opening and brake pedal opening. These inputs, together with real-time vehicle status information, are used to predict key output variables such as vehicle acceleration, instantaneous engine fuel consumption, and battery power consumption.

The modeling framework comprises three sequential components:

1. **Pedal MAP Module:** This initial stage processes the accelerator and brake pedal inputs alongside current vehicle speed to estimate the required driving and braking forces.
2. **Energy Management Strategy:** Based on the calculated force demand and real-time battery SOC, this stage optimally distributes torque among the engine, motor, and mechanical braking system.
3. **Vehicle Dynamics Model:** This final stage computes vehicle acceleration in response to the applied forces. The engine module estimates fuel consumption using torque and rotational speed data, while the motor and battery modules calculate power consumption by incorporating torque, speed, and internal resistance characteristics.

The model operates in a closed-loop configuration: vehicle acceleration is integrated to obtain speed, and battery power consumption is accumulated to update the SOC. Both updated values are then fed back as inputs to maintain dynamic consistency across time steps. Furthermore, the predicted fuel and electric power consumption serve as key performance metrics for assessing the overall energy efficiency of the vehicle under different driving conditions.

Building this model using traditional physics-based methods is impractical. It would require secret vehicle control strategies and detailed powertrain data from the manufacturer, which are hard to obtain. The pedal map, often referred to as the “DNA” of a vehicle, is essential for providing a unique driving experience. It encapsulates the manufacturer’s design philosophy and tailored interpretation of vehicle performance characteristics. Simultaneously, the energy management strategy plays a pivotal role in enabling PHEVs to outperform conventional ICEVs in terms of energy efficiency. This strategy governs the optimal allocation of energy demands across various powertrain components and serves as the central determinant of overall vehicle energy utilization. However, due to the complexity and sheer number of parameters involved in modern powertrain systems, it is neither feasible nor cost-effective to obtain complete parameter sets during vehicle integration or calibration stages. As a result, relying on traditional physics-based modeling approaches becomes increasingly impractical, particularly when aiming to capture real-world operational dynamics and evolving system behaviors.

In addition, powertrain-related parameters are typically obtained through component-level testing conducted prior to the initiation of new vehicle development. In contrast, vehicle-level parameters are measured during prototype testing phases. Both types of data reflect the vehicle’s performance in its as-new condition. However, in real-world operation, significant aging effects manifest in key components such as the transmission system and the battery [26,27]. For instance, the transmission system may experience slight efficiency improvements due to gear break-in effects, resulting in approximately a 2% reduction in energy consumption over time. On the other hand, battery degradation leads to increased internal resistance and reduced capacity, which collectively decrease the available electric energy by around 4.8%. These aging phenomena are highly dependent on actual vehicle usage patterns. Consequently, even vehicles of the same model and configuration can exhibit notable differences in energy consumption and emissions after being subjected to varying driving conditions.

Given the aforementioned limitations of traditional modeling approaches, we propose a data-driven methodology for developing a driver behavior-based PHEV model. This approach makes it easier to construct an end-to-end vehicle model. Driver behaviour signals, such as accelerator and brake pedal inputs, are used as inputs, and key dynamic and energy-related outputs, including vehicle acceleration, fuel consumption and battery power, are predicted accordingly. The data-driven model is developed using real-world experimental data and trained through a carefully designed neural network architecture. Throughout the training process, the model progressively learns the functional mapping from input features to target outputs, aligning its predictions with the patterns observed in empirical data. This learning-based approach effectively replaces the need for explicit physical modeling, allowing the system to capture complex behaviors directly from data.

There are multiple advantages to building this data-driven model:

- Firstly, it eliminates the need for an in-depth understanding of the complex physical mechanisms underlying intermediate processes by focusing solely on the relationship between inputs and outputs.
- Secondly, the accuracy of the model depends only on the quality of the collected data and the suitability of the network architecture. Both of these factors can be assessed quantitatively, thus avoiding the uncontrollable accuracy issues that are often introduced by simplifications in physics-based models.
- Thirdly, the model can be updated as new experimental data becomes available, enabling it to capture the inherent degradation characteristics of the vehicle system over time.
- Fourthly, with sufficient test data, this modelling method can be rapidly extended to any vehicle type, making it highly suitable for large-scale vehicle modelling applications.

These advantages make the data-driven approach not only more practical and flexible than traditional methods but also highly effective in capturing the nonlinear, multi-mode relationships between driver behavior and vehicle energy consumption.

## 3 Model architecture

Developing an accurate PHEV model grounded in driver behavior fundamentally hinges on the design of an effective training architecture. In the following sections, we provide a comprehensive description of the proposed DLLT framework, focusing on three key components: model inputs and outputs, PHEV mode classification, and the dual-layer model training architecture.

### 3.1 Model inputs and outputs

When analyzing the influence of driver behavior on vehicle energy consumption, various behavioral and dynamic factors can be considered, such as vehicle speed, acceleration, deceleration, pedal operations, gear shifting, and steering behavior [28]. Among these, vehicle speed is a foundational variable in energy consumption modeling and plays a critical role in capturing driving dynamics. While acceleration and deceleration reflect the operational states of the accelerator and brake pedals, respectively, pedal signals provide a more direct representation of the driver’s control intent. Consequently, this study selects the accelerator pedal opening (AccPedal) and brake pedal opening (BrakePedal) as primary input features. For PHEVs, the structural design eliminates the need for conventional gear-shifting mechanisms. As a result, gear-related parameters are not considered in this study. However, PHEVs are equipped with rechargeable energy storage systems, and the battery SOC plays a significant role in determining overall energy consumption. Therefore, SOC is incorporated as a key input variable in the model. Moreover, during highway driving scenarios, the additional resistance induced by steering operations is relatively small and has minimal impact on total energy consumption [29]. Consequently, driver steering behavior is excluded from the input feature set. To minimize the influence of environmental variables on the experimental outcomes, all tests are conducted under controlled laboratory conditions. Specifically, real-vehicle testing is performed on an indoor chassis dynamometer, with road grade fixed at $0^{\circ }C$, ambient temperature maintained at $25^{\circ }C$, wind speed set to zero, and the air conditioning system deactivated throughout the experiment. These controlled settings help reduce external environmental interference and ensure consistent energy consumption measurements.

In summary, we develop a neural network model that incorporates driver operational behavior and vehicle status as primary input variables. The selected input features include AccPedal, BrakePedal, Velocity, and battery SOC. The output layer consists of both dynamic and energy-related performance indicators, specifically: vehicle acceleration (Acceleration), instantaneous engine fuel consumption rate (Fuel Consumption, FC), and battery power (ePower). Together, these outputs capture the vehicle’s dynamic responses and energy consumption patterns across diverse driving behaviors.

### 3.2 PHEV mode analysis

Unlike conventional ICEVs, which rely exclusively on the engine for propulsion, or BEVs, which are fully dependent on the electric motor, PHEVs incorporate a sophisticated multi-source powertrain architecture. This design enables PHEVs to operate across multiple driving and braking modes depending on real-time driving conditions. The multi-mode capability of PHEVs enhances their adaptability in energy management while maintaining high dynamic performance, ultimately improving overall energy utilization efficiency. However, this increased operational flexibility also introduces a more complex interplay between energy consumption patterns and driver behavior, making it significantly more challenging to model compared to vehicles powered by a single energy source.

**Fig 3:**
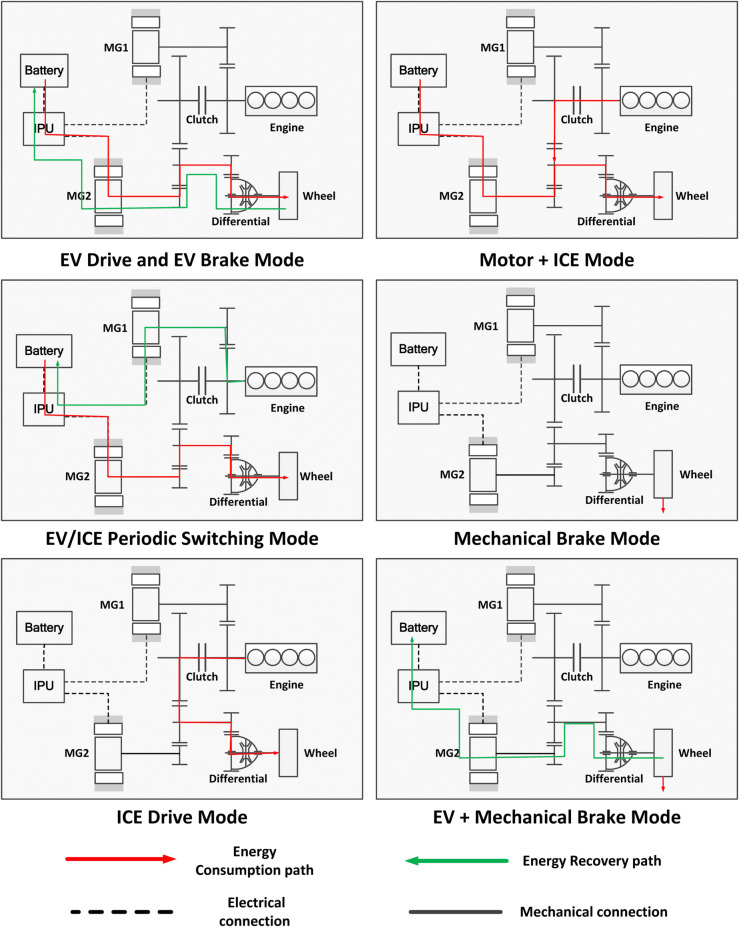
Operating modes of PHEVs.

To gain a deeper understanding of how driver inputs influence vehicle dynamics and energy consumption across different operating modes, this study systematically categorizes the main driving and braking modes into distinct classes. Fig 3 presents the various operational modes of PHEVs.

1. **EV Drive Mode (Battery Electric Drive):**In this mode, the vehicle is solely powered by the electric motor. When the driver presses the accelerator pedal, the input signal is processed by the motor controller and converted into a corresponding torque demand that drives the wheels. The resulting acceleration behavior closely resembles that of EVs, with continuous battery power consumption occurring throughout operation. This mode is predominantly utilized in low-speed, low-load urban driving conditions, where energy demand is relatively modest and electric propulsion is most efficient.
2. **Motor + ICE Mode (Hybrid Drive):** In this mode, both the internal combustion engine and the electric motor contribute simultaneously to vehicle propulsion. The driver’s acceleration demand is distributed between the two power sources based on the predefined energy management strategy, leading to a combined torque output that drives the wheels. The vehicle demonstrates dynamic response characteristics comparable to those of conventional HEVs, with concurrent changes in engine fuel consumption and battery power usage. This mode is typically engaged during medium- to high-speed driving or under high-load conditions, where the combined power output from both energy sources ensures optimal performance and efficiency.
3. **EV/ICE Periodic Switching Mode (Periodic Mode Switching):** In this mode, the PHEV dynamically alternates between pure electric propulsion and engine-based driving. This switching is guided by both real-time driving conditions and the energy management strategy. Depending on the current sub-mode, the vehicle’s response to the driver’s acceleration input is delivered either through the electric motor or the internal combustion engine. In some cases, the generator may also be engaged for onboard power generation. Consequently, the vehicle’s dynamic performance exhibits characteristics of both electric and hybrid vehicles. Both engine fuel consumption and battery power consumption display periodic variations, reflecting the complex and nonlinear nature of the system’s energy flow.
4. **ICE Drive Mode (Engine-Only Drive):** In this mode, the internal combustion engine functions as the exclusive power source, directly propelling the vehicle’s wheels. The driver’s acceleration command is conveyed through throttle control and the transmission system. This generates dynamic performance comparable to conventional ICEVs, accompanied by elevated fuel consumption. This mode is activated under two typical scenarios: when the battery SOC falls below a predefined threshold, or during high-load driving conditions where the demand exceeds the electric motor’s capacity. In such cases, the engine assumes full responsibility for propulsion.
5. **EV Brake Mode (Regenerative Braking Only):** In this mode, the vehicle primarily utilizes the electric motor to generate braking torque, facilitating both deceleration and kinetic energy recovery. The driver’s brake pedal input is interpreted by the motor controller and translated into a corresponding level of regenerative braking intensity. This directly influences the vehicle’s deceleration behavior and the amount of energy returned to the battery. Mechanical friction braking is seldom activated in this mode, resulting in minimal energy loss and high regenerative efficiency. This mode is typically engaged during light or moderate braking events, such as when decelerating on flat roads or approaching traffic signals with sufficient lead time.
6. **EV + Mechanical Brake Mode (Combined Regenerative and Friction Braking):** In this mode, the driver’s braking input is distributed between the motor-based regenerative braking system and the mechanical braking system. The vehicle combines both mechanisms to achieve controlled deceleration. Each subsystem contributes to the total braking force applied to the wheels. This affects both the overall deceleration performance and the amount of energy recovered. This hybrid strategy is typically engaged during moderate to heavy braking events. It strikes a balance between ensuring braking safety and maximizing energy recuperation.
7. **Mechanical Brake Mode (Friction Braking Only):** In this mode, the car uses only its traditional hydraulic brakes, with no energy recovery. When you press the brake pedal, hydraulic pressure clamps the brake discs to slow the car down. Because no energy is recaptured, the battery isn’t recharged. This mode is used for safety-critical stops, like emergencies, or when the electric motor can’t assist. It guarantees reliable braking but wastes the opportunity to save energy.

It is important to acknowledge the significant variations in the nonlinear relationships between energy consumption characteristics and driver behavior, vehicle speed, and battery SOC across different operational modes. Furthermore, during transitions between these modes, sudden fluctuations in energy consumption and power output frequently occur due to physical inertia within the powertrain system and delays in control logic switching. In the event of neural network models being trained exclusively on data from a single mode, there is a possibility that they may suffer from reduced generalization capability during mode transitions. This may result in inaccurate predictions of vehicle responses under new or unseen operating conditions.

In order to address this limitation and enhance the model’s adaptability and robustness, it is imperative to incorporate the “vehicle operating mode” as a pivotal input feature within the neural network framework, thereby reflecting the current configuration of the powertrain system. It is acknowledged that the operating mode is determined by driver behavior, vehicle speed, and battery SOC. The present study proposes a data-driven clustering approach to automatically identify and extract representative operating mode categories from historical driving data. The integration of these prediction mode features into the model inputs facilitates enhanced accuracy in modeling and prediction of multi-mode energy consumption dynamics.

### 3.3 PHEV model training architecture

**Fig 4:**
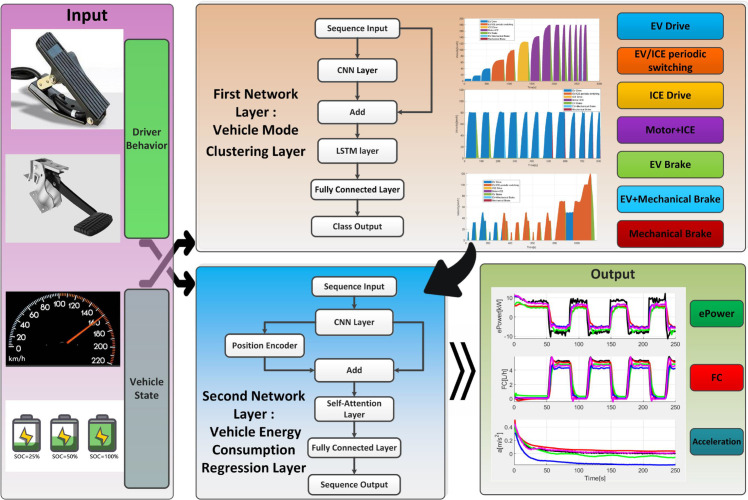
Training framework of the DLLT model.

This paper proposes a multi-mode PHEV eco-driving behavior model based on a dual-layer LSTM-Transformer training architecture, named DLLT. As illustrated in Fig 4, the model consists of two hierarchical layers. The first layer, called the vehicle mode clustering layer, takes the vehicle information as inputs. These features are fed into an LSTM network to identify the specific driving mode that the vehicle is operating in during each time period. The second layer, called the vehicle energy consumption regression layer, utilizes both the mode identification results from the first layer and the original input variables as features. These are fed into a Transformer network to predict key vehicle state parameters. By effectively integrating multi-dimensional real-time vehicle data, DLLT enables high-precision modeling and prediction of vehicle operational states under complex driving scenarios, thereby enhancing the accuracy and robustness of eco-driving behavior recognition. The following sections will provide a detailed introduction to the model architecture and key technical details of the DLLT model.

### 3.4 Layer 1: Vehicle mode clustering layer

The core objective of this layer is to identify and cluster the operational modes of PHEVs across different time periods. Given that PHEVs are capable of multi-mode propulsion and their operational modes dynamically change over time, a model capable of effectively capturing temporal dependencies is required for accurate mode recognition. This layer employs a LSTM network to model the input time series data. The input features consist of four key variables: AccPedal, BrakePedal, Velocity, and SOC. These variables collectively provide a comprehensive representation of driver behavior, the vehicle’s current operating status, and the influence of energy management strategies on the overall system.

LSTM is an improved variant of recurrent neural networks (RNNs) that addresses the issues of gradient vanishing and long-term dependency commonly encountered in traditional RNNs by incorporating gating mechanisms [30]. This architecture renders LSTM particularly well-suited for processing driving cycle data with complex temporal characteristics, thereby enabling more accurate capture of mode transition patterns under diverse driving scenarios in PHEVs. The specific formulas for LSTM are shown below.

$$f_{t}=\sigma (W_{f}\cdot [h_{t},x_{t}]+b_{f})$$ (1)

$$i_{t}=\sigma (W_{i}\cdot [h_{t},x_{t}]+b_{i})$$ (2)

$$\tilde{C_{t}}=\sigma (W_{C}\cdot [h_{t},x_{t}]+b_{C})$$ (3)

$$C_{t}=f_{t}⊙C_{t-1}+i_{t}⊙\tilde{C_{t}}$$ (4)

$$o_{t}=\sigma (W_{o}\cdot [h_{t},x_{t}]+b_{o})$$ (5)

$$h_{t}=o_{t}⊙tanh(C_{t})$$ (6)

In the LSTM network, the hidden state and cell state at each time step *t* are updated through three key gating mechanisms: the forget gate, the input gate, and the output gate. Specifically, Formula 1 corresponds to the forget gate, Formula 2 to the input gate, and Formula 5 to the output gate. At time step *t*, the input vector is denoted as *x*_*t*_, while the hidden state and cell state are denoted as *h*_*t*_ and *C*_*t*_, respectively. The weight matrices and bias terms are represented by W and b, respectively. The activation functions employed include the sigmoid function *σ* and the hyperbolic tangent function *tanh*, which introduce non-linearity into the gating and state update processes.

Through the aforementioned recursive updating mechanism, the LSTM network is capable of constructing time-dependent hidden representations progressively based on historical data, without relying on future information. This capability enhances the identification of various operational modes of PHEVs in complex driving environments, including EV Drive, Motor+ICE, EV/ICE cyclic switching, ICE Drive, EV Brake, EV+Mechanical Brake, and Mechanical Brake. Furthermore, it provides critical mode-specific features that support subsequent energy consumption regression analysis.

### 3.5 Layer 2: Vehicle energy consumption regression layer

The second layer is the vehicle energy consumption regression layer, whose primary objective is to predict the instantaneous energy consumption metrics of the PHEV based on the previously identified operational modes. These metrics include *FC*, *ePower*, and *Acceleration*. This layer employs the Transformer network as the primary modeling framework, which takes both the mode flags output from the first layer and the original input features, i.e., accelerator pedal opening, brake pedal opening, vehicle speed, and battery SOC, to achieve accurate energy consumption prediction.

The Transformer is a deep learning architecture based on the self-attention mechanism, initially developed for natural language processing tasks [31]. By incorporating positional encoders and self-attention mechanisms, the Transformer can effectively capture long-range dependencies without relying on recurrent computations, while also enabling parallel processing to significantly improve training efficiency. This capability is particularly valuable in the energy consumption modeling of PHEVs, where complex interactions among different power sources across various driving modes require global contextual integration. The formulas for position encoding are as follows:

$$PE_{\left(pos,2i\right)}=sin(\frac{pos}{10000^{\frac{2i}{d_{model}}}})$$ (7)

$$PE_{\left(pos,2i+1\right)}=cos(\frac{pos}{1000^{\frac{2i}{d_{model}}}})$$ (8)

In the formulas, *pos* denotes the position of the current time step within the sequence, *i* is the dimension index of the input feature vector, and *d*_*model*_ represents the dimensionality of the input features. The positional encoding *PE* is generated using sine and cosine functions to produce periodic encoding values. This enables the model to distinguish between input features at different time steps and preserve the sequential order of the time series data. Such a design enhances the model’s robustness and generalization capability when capturing long-term dependencies.

**Fig 5:**
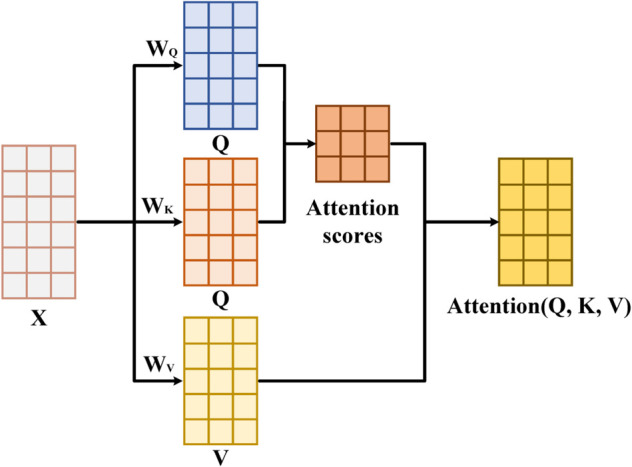
The computational workflow of the self-attention mechanism.

The self-attention mechanism is the core component of the Transformer architecture. The computational workflow is illustrated in Fig 5. It enables the model to identify which input features have the most significant impact on the current output by computing the correlation weights among all features. The formulas for self-attention are shown below.

$$Q=XW_{Q},\;K=XW_{K},\;V=XW_{V}$$ (9)

$$Attention(Q,K,V)=softmax(\frac{QK^{T}}{\sqrt{d_{k}}})V$$ (10)

The three key vectors, Query (*Q*), Key (*K*), and Value (*V*) are first obtained through linear transformations of the input. The dot product between *Q* and *K* is then used to measure the relevance between different features, and the resulting attention weights are applied to *V* to produce a weighted sum that forms the final output. Through this mechanism, the model can dynamically capture the interactions among different power sources—such as the internal combustion engine (ICE), electric motor, and mechanical braking system—under various operational modes. Moreover, it allows the model to automatically identify which features contribute most to energy consumption in each mode, enabling adaptive adjustment of the prediction strategy based on contextual information.

To further enhance the model’s representational capacity and generalization performance, the Transformer architecture introduces the Multi-Head Attention mechanism. This mechanism employs multiple distinct attention heads to compute attention weights in parallel, allowing the model to focus on different aspects of the input simultaneously. The outputs from these heads are then concatenated and linearly projected back to the original dimension. The computation can be formulated as follows.

$$MultiHead(Q,K,V)=Concat(head_{1},head_{2},...,head_{h})W_{O}$$ (11)

$$head_{i}=Attention(Q,K,V)$$ (12)

This multi-head design enables the model to extract information from multiple subspaces, thereby capturing diverse perspectives of the input features. It significantly improves the model’s ability to characterize complex driving behaviors and the intricate relationships in energy allocation, especially during mode transitions. By learning representations that account for the nonlinear dynamics and latent interactions among different operational modes, the Multi-Head Attention mechanism enhances the model’s sensitivity to subtle yet critical changes in the vehicle’s energy consumption patterns.

## 4 Experiment

**Fig 6:**
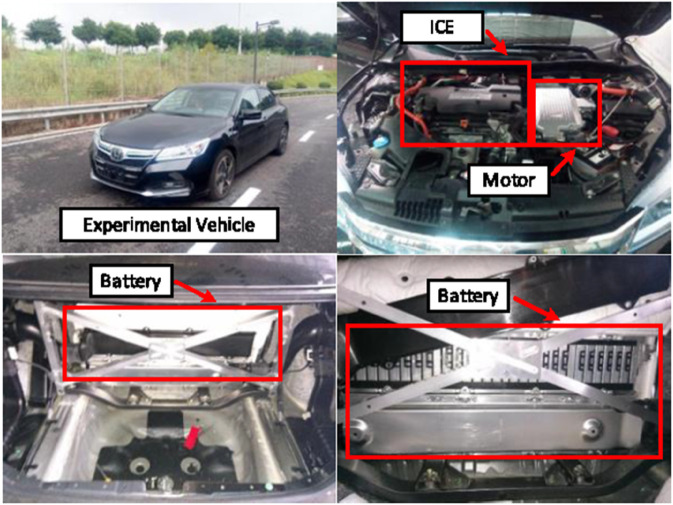
The powertrain configuration of the experimental vehicle platform.

Following the completion of the DLLT training architecture design, DLLT is implemented and trained using real-world driving data. The experiment was conducted using a Honda Accord PHEV as the experimental vehicle platform. The powertrain configuration of the experimental vehicle is illustrated in Fig 6. All powertrain components, including the internal combustion engine, generator, traction motor, and their respective controllers, are integrated within the front compartment of the vehicle. High-voltage components such as the lithium-ion battery pack, battery management system, and relays are centrally located in the trunk area. The battery enclosure is designed with a two-tier structure, comprising upper and lower layers, to optimize space utilization and thermal management. By iteratively refining the network parameters, DLLT learns the complex dynamics of vehicle behavior.

The dataset details are presented in Table 1, comprising a total of 2,415,000 data points collected at a sampling interval of 0.01 seconds, resulting in a total driving duration of 24,150 seconds. Among this, 18,260 seconds of data were used for model training, covering single acceleration events (divided into two groups based on initial battery State of Charge (SOC) at 30% and 80%), single braking events, and multiple cyclic operating conditions (including 4 JC08, 3 HWY, and 3 UDDS cycles). The data for validation and testing consisted of 5 NEDC driving cycles, with a total duration of 5,890 seconds. All cyclic condition tests commenced with the battery SOC at 100% and continued until the charge-sustaining phase was reached. The time distributions of the training, validation, and test sets across different driving modes are detailed in Table 2 and Table 3, respectively.

**Table 1:** Overview of data collection conditions and dataset partition.

No.	Test type	Control pedal	Pedal quantity (%)	SOC(%)	Test time (s)	Dataset split
1	Single acceleration	Acceleration pedal	0; 5; 10; 15; 20; 25; 30; 35; 40; 50; 60; 70; 80; 90; 100	Initial: 30%	2743	Train (18260s)
			5; 10; 15; 20; 25; 30; 40; 50; 60; 70; 80; 90; 100	Initial: 80%	2333	
2	Single braking	Brake pedal	0; 3; 5; 7; 10; 12; 15; 20; 25; 30; 35; 40; 45; 50	Initial: 30%	1693	
3	Cyclic operation condition	Following vehicle speed	4 JC08	Continuously decrease from 100% to 30%	5087	
			3 HWY		2361	
			3 UDDS		4043	
			5 NEDC		5890	validation/test (5890s)
Total Driving Time					24150s	

**Table 2:** Distribution of operating modes in the training data.

Mode	EV Drive	EV/ICE periodic switching	ICE Drive	Motor+ICE	EV Brake	EV + Mechanical Brake	Mechanical Brake	Stop
Time(s)	6109	4327	237	2499	1926	256	27	2879
Percentage(%)	33.46	23.7	1.3	13.69	10.55	1.4	0.15	15.77

**Table 3:** Distribution of Operating Modes in validation and test data.

Mode	EV Drive	EV/ICE periodic switching	ICE Drive	Motor+ICE	EV Brake	EV + Mechanical Brake	Mechanical Brake	Stop
Time(s)	2011	1595	0	269	700	35	0	1280
Percentage(%)	34.14	27.08	0	4.57	11.88	0.59	0	21.73

### 4.1 Performance metric

In the vehicle mode clustering layer for PHEVs, the primary goal is to identify the vehicle’s operational mode at each time step. To assess the performance of this layer, we employ classification accuracy as the evaluation metric. Accuracy is calculated as the proportion of correctly classified samples relative to the total number of test instances, offering a straightforward measure of the model’s capability to differentiate between distinct driving modes.

In contrast, the vehicle energy consumption regression layer focuses on modeling and predicting energy consumption at each time step, based on the identified operational modes and historical driving data. Given the nature of this task as a regression problem, its performance is assessed using a comprehensive set of evaluation metrics: Mean Absolute Error (MAE), Mean Absolute Percentage Error (MAPE), Mean Squared Error (MSE), Root Mean Squared Error (RMSE), Normalized Root Mean Squared Error (NRMSE), and the coefficient of determination (*R*^2^). These metrics provide a multi-dimensional assessment of the model’s predictive performance, capturing aspects such as absolute error magnitude, relative error, model stability, and overall goodness of fit.

$$Accuracy=\frac{n_{Class\_Prediction\;==\;Class\_True}}{n}\times 100\%$$ (13)

$$MAE=\frac{1}{n}\sum_{i=1}^{n}|y_{i}-\hat{y_{i}}|$$ (14)

$$MAPE=\frac{1}{n}\sum_{i=1}^{n}|\frac{y_{i}-\hat{y_{i}}}{y_{i}}|$$ (15)

$$MSE=\frac{1}{n}\sum_{i=1}^{n}(y_{i}-\hat{y_{i}}{)}^{2}$$ (16)

$$RMSE=\sqrt{\frac{1}{n}\sum_{i=1}^{n}(y_{i}-\hat{y_{i}}{)}^{2}}$$ (17)

$$Normalized\;RMSE\;(NRMSE)=\frac{\sqrt{\frac{1}{n}\sum_{i=1}^{n}(y_{i}-\hat{y_{i}}{)}^{2}}}{y_{max}-y_{min}}$$ (18)

$$R^{2}=1-\frac{\sum_{i=1}^{n}(y_{i}-\hat{y_{i}}{)}^{2}}{\sum_{i=1}^{n}(y_{i}-\bar{y_{i}}{)}^{2}}$$ (19)

Here, *i* denotes each individual time step, and *n* represents the total number of time steps. $n_{Class_{P}rediction=Class_{T}rue}$ indicates the number of samples for which the predicted class matches the true class. *y*_*i*_ denotes the actual output value at the *i*-th time step, $\hat{y^{i}}$ represents the corresponding predicted output value, and $\bar{y}$ stands for the mean of all actual output values.

Among the aforementioned metrics, higher classification accuracy (closer to 100%) indicates superior performance in identifying vehicle operation modes. For regression tasks, lower values of MAE, MAPE, MSE, RMSE, and NRMSE correspond to reduced prediction errors, whereas an *R*^2^ value approaching 1 demonstrates a strong alignment between predicted and actual outputs.

However, due to the presence of numerous zero values in the measured *ePower*, *FC*, and *Acceleration* data, the computation of MAPE often results in infinite or undefined values. As a result, MAPE is not adopted as an evaluation metric for assessing the performance of the neural network in this study.

### 4.2 Hyperparameter optimization

When developing high-performance deep learning models, the judicious configuration of hyperparameters is paramount in determining training efficiency, convergence behavior, generalization capability, and ultimately predictive accuracy. However, traditional hyperparameter tuning approaches such as manual trial-and-error, grid search or random search often incur high computational costs and have low search efficiency. They also tend to converge to suboptimal solutions, particularly when dealing with high-dimensional and nonlinear hyperparameter spaces. These limitations make identifying globally optimal configurations challenging, especially as model complexity increases.

To address these challenges, this study employs the Northern Goshawk Optimization (NGO) algorithm to automate the hyperparameter tuning process for the proposed DLLT model as well as several baseline architectures [32]. NGO is a nature-inspired metaheuristic optimization algorithm that emulates the hunting behavior of the Northern Goshawk, including its strategies for exploration, recognition, pursuit, and adaptive evasion. By balancing global exploration and local exploitation, NGO demonstrates strong optimization capabilities, rapid convergence, and robust performance across complex search landscapes. The algorithm has shown promising results in various engineering and machine learning applications. In this work, NGO is systematically applied to optimize the key hyperparameters of each model. Tables 4 and 5 summarize the resulting optimized parameter configurations in DLLT and baselines.

**Table 4:** Optimized hyperparameter configurations for DLLT.

Parameters	LSTM	Parameters	Transformer		
Output	Mode	Output	Accel.	ePower	FC
Number of Layers	12	number of layers	14		
activation functions	ReLU	activation functions	ReLU		
number of attention heads	-	number of attention heads	1	1	1
hidden units	50	number of attention keys	29	22	23
feed-forward dimensions	8	Embedding Output Size	464		
learning rate	0.01	learning rate	$1\times 10^{-3}$	$2.5\times 10^{-3}$	$1.0\times 10^{-3}$
optimizer	Adam	optimizer	Adam		

**Table 5:** Optimized hyperparameter configurations for baseline models.

Model	LSTM			GRU			CBA			Transformer		
Output	ePower	FC	Accel.	ePower	FC	Accel.	ePower	FC	Accel.	ePower	FC	Accel.
Learning Rate ($1\times e^{-3}$)	8.4	8.0	5.9	1.9	3.5	8.6	5.9	5.1	3.5	2.0	3.9	2.2
Hidden Units	37	118	54	35	46	150	22	11	43	44	44	44
Key Dimension Per Head	-	-	-	-	-	-	27	20	13	29	11	4
Normalizing Parameter ($1\times e^{-4}$)	6.1	8.1	3.3	3.1	3.8	3.7	4.2	6.7	5.4	1.4	2.9	1.0

### 4.3 PHEV mode analysis

**Fig 7:**
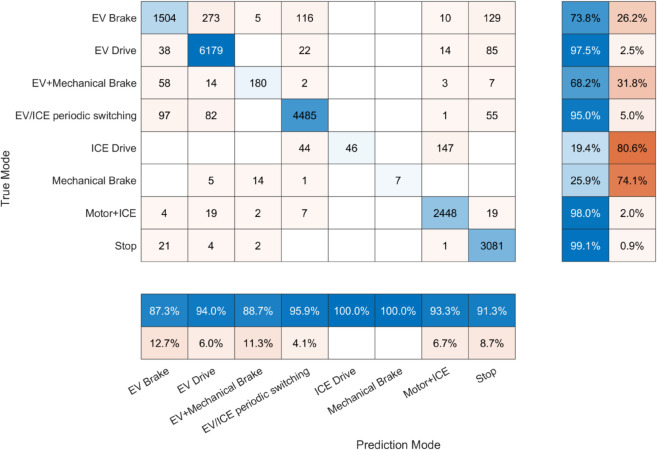
Confusion matrix for mode prediction performance.

The confusion matrix illustrating the performance of mode prediction is presented in Fig 7. In this matrix, the vertical axis denotes the actual operational modes, while the horizontal axis indicates the corresponding predicted modes. Each cell value reflects the number of instances associated with a specific combination of true and predicted modes, with the diagonal entries representing the count of correctly classified samples. In the rightmost two columns of the matrix, the proportions of correctly predicted and misclassified samples are displayed for each true mode. Similarly, the bottom two rows provide the accuracy and misclassification rates for each predicted mode, offering a comprehensive view of the model’s classification behavior from both perspectives.

The overall classification accuracy achieved by the vehicle mode clustering layer in the PHEV mode identification task reaches 93.23%. Among the true modes, EV Drive, EV/ICE periodic switching, Motor+ICE, and Stop demonstrate the highest recognition rates, all surpassing 95%. Moreover, in terms of predicted modes, ICE Drive and Mechanical Brake exhibit the lowest cross-mode misclassification rates, reflecting strong discriminative capability and high specificity in identifying these operation states.

**Fig 8:**
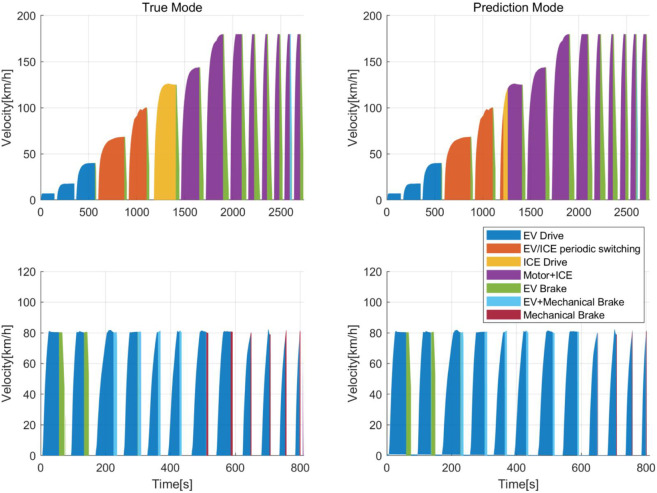
Comparison of true and predicted mode velocity time series.

As illustrated in Fig 8, a temporal comparison between the ground truth and predicted modes demonstrates that the model effectively captures PHEV operational modes using acceleration data, with only a limited number of misclassifications, primarily involving the confusion between ICE Drive and Motor+ICE modes. During the braking phase, the model occasionally misidentifies the Mechanical Brake mode as EV+Mechanical Brake. Nevertheless, the vehicle mode clustering layer achieves a high level of accuracy in mode identification, providing robust and reliable mode features for the subsequent energy consumption regression layer.

### 4.4 PHEV energy analysis

To comprehensively evaluate the performance of different neural network architectures in modeling multi-mode energy-saving driving behaviors of PHEVs, this study conducts comparative experiments among commonly used temporal data training architectures. These include LSTM, Gated Recurrent Unit (GRU), Convolutional Neural Network + Bidirectional LSTM + Self-Attention (CBA), and Transformer, alongside the proposed two-stage LSTM-Transformer architecture. The predictive capabilities of these models on key vehicle energy-related variables, including *ePower*, *FC*, and *Acceleration*, are assessed using multiple performance metrics such as *MAE*, *MSE*, *RMSE*, and *R*^2^.

As illustrated in Table 6, a comprehensive overview of the performance metrics for each model is provided. As demonstrated, DLLT consistently exhibits superior performance in comparison to all baseline architectures across the full range of evaluation criteria pertaining to battery power prediction. The model achieves an *MAE* of 3.2941, an *MSE* of 31.7381, an *RMSE* of 5.6337, an NRMSE of 0.0448, and an *R*^2^ score of 0.8053, thereby clearly demonstrating its superior predictive accuracy and generalization ability. With regard to the prediction of engine fuel consumption, DLLT demonstrates excellent performance with an *MAE* of 0.3747, *MSE* of 0.3800, *RMSE* of 0.6165, *NRMSE* of 0.014, and a remarkably high *R*^2^ of 0.9945, thereby significantly surpassing the performance of other models and highlighting its exceptional ability to capture fuel consumption dynamics. With regard to the task of vehicle acceleration prediction, the DLLT model demonstrates the highest *R*^2^ value of 0.86, thus exhibiting a significant degree of superiority in relation to the other models. This finding suggests that DLLT architectures are the most effective in capturing the dynamic characteristics of vehicle motion. The experimental results obtained demonstrate the validity of the effectiveness and robustness of DLLT in modelling complex, multi-mode driving behaviours for PHEVs.

**Table 6:** Performance comparison of different models for PHEV energy consumption and driving dynamics prediction.

Output	Metrics	LSTM	GRU	CBA	Transformer	DLLT
ePower	MAE	4.3410	4.1483	3.6023	3.4493	3.2941
	MSE	44.7374	41.8981	35.4988	32.8368	31.7381
	RMSE	6.6886	6.4729	5.9581	5.7303	5.6337
	NRMSE	0.0532	0.0515	0.0474	0.0456	0.0448
	*R* ^2^	0.7255	0.7430	0.7822	0.7985	0.8053
FC	MAE	0.9345	0.9737	0.5408	0.5283	0.3747
	MSE	1.7719	2.0162	0.7775	0.9693	0.3800
	RMSE	1.3311	1.4199	0.8818	0.9845	0.6165
	NRMSE	0.0303	0.0323	0.0201	0.0224	0.014
	*R* ^2^	0.9744	0.9709	0.9888	0.9860	0.9945
Acceleration	MAE	0.2147	0.3176	0.2228	0.1104	0.1092
	MSE	0.1284	0.2203	0.0968	0.0409	0.0423
	RMSE	0.3583	0.4693	0.3111	0.2022	0.2057
	NRMSE	0.033	0.0432	0.0286	0.0186	0.0189
	*R* ^2^	0.5646	0.2528	0.6716	0.8613	0.8565

**Fig 9:**
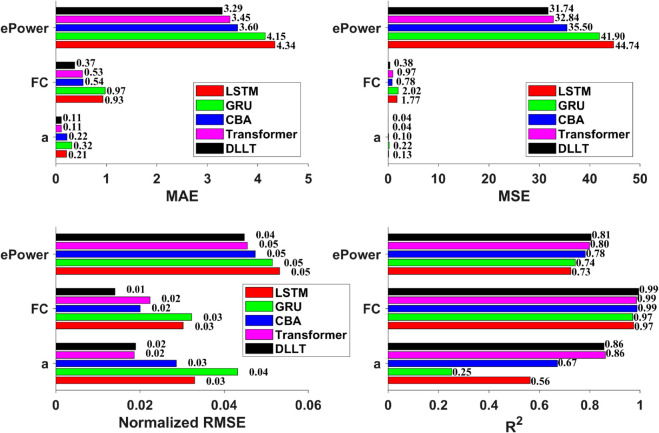
The performance of different models across various evaluation metrics.

Fig 9 further illustrates the performance of different models across various evaluation metrics. For *ePower* prediction, the ranking from best to worst is: DLLT > Transformer > CBA > GRU > LSTM. In the case of FC, the order is: DLLT > CBA > Transformer > LSTM > GRU. As for Acceleration, both DLLT and Transformer achieve comparable and superior performance over the other models. Moreover, the *R*^2^ values further confirm the superiority of the DLLT model. It achieves an *R*^2^ of 0.99 for FC, 0.81 for *ePower*, and 0.86 for *Acceleration*, all of which outperform the other architectures, demonstrating its strong predictive capability and robustness.

**Fig 10:**
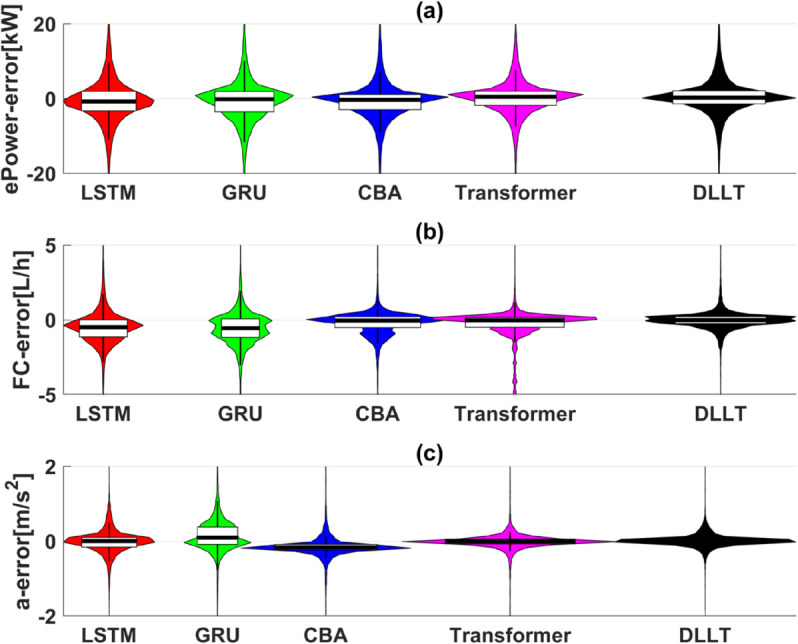
Error distribution analysis of different models.

Fig 10 compares the error distribution among different models. For *ePower*, the errors of DLLT, Transformer, and CBA are more tightly distributed around zero, with the DLLT showing the most symmetric error distribution, indicating superior stability. Regarding FC prediction, the DLLT exhibits the smallest error, closely centered around zero, while GRU and LSTM show larger negative biases. In terms of acceleration prediction, the DLLT and Transformer again demonstrate the tightest and most centralized error distributions around zero, reflecting their stronger predictive accuracy compared to other models.

**Fig 11:**
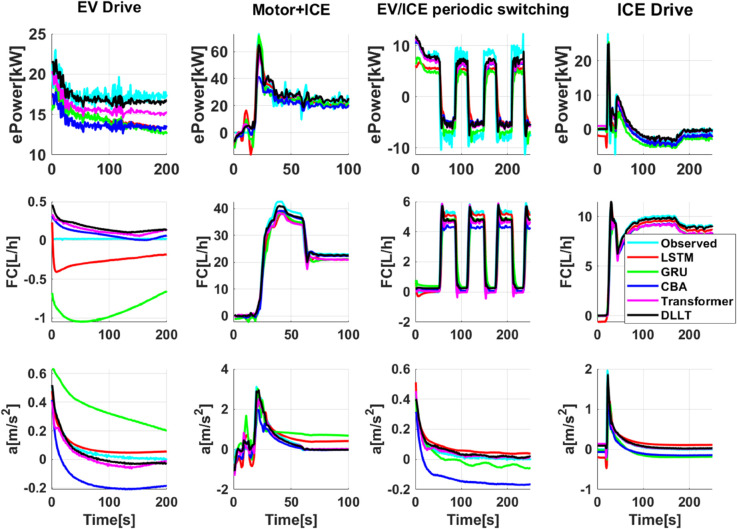
Comparison of PHEV energy and dynamics prediction under different driving modes.

**Fig 12:**
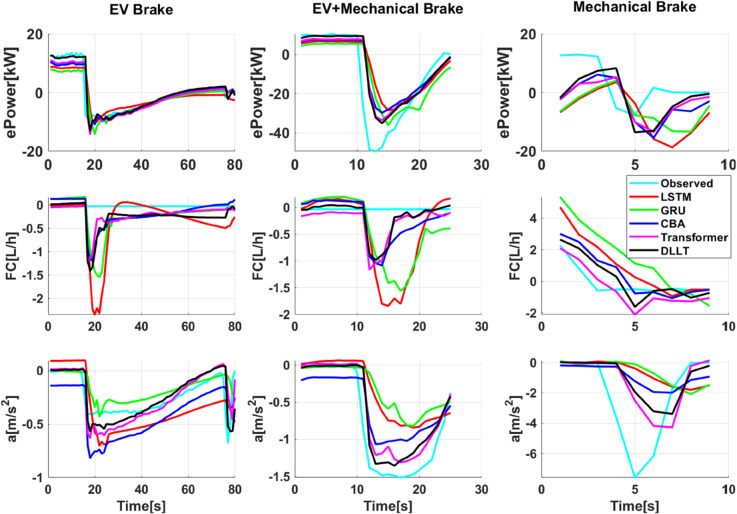
Comparison of PHEV energy and dynamics prediction under different braking status.

Further analysis based on different driving and braking modes (illustrated in Figs 11 and 12) demonstrates that the DLLT achieves the highest prediction accuracy under modes such as Motor+ICE, EV/ICE periodic switching, and ICE Drive. It effectively captures the dynamic trends in battery power and engine fuel consumption, and also shows good consistency in acceleration prediction. The model maintains high accuracy in EV Drive and EV Brake modes as well. However, in the EV+Mechanical Brake and Mechanical Brake modes, where mechanical braking dominates and motor activity is reduced, the predictions of electric power and fuel consumption lag behind the actual values, leading to relatively weaker performance in these specific conditions.

In summary, the multi-mode PHEV eco-driving behavior model based on DLLT dual-layer architecture outperforms existing methods in overall energy consumption and dynamic performance prediction. Particularly in scenarios involving engine-driven operation and motor-dominated braking, the model demonstrates higher accuracy and stability. These results indicate that the DLLT architecture is capable of effectively capturing the complex energy distribution relationships and temporal dependencies inherent in PHEVs, offering strong practical applicability and potential for broader implementation.

In addition, the above data analysis reveals that the predictive performance of the multi-mode PHEV eco-driving behavior model varies across different driving modes. The highest prediction accuracy is achieved in the Motor+ICE, EV/ICE periodic switching, and ICE Drive modes, followed by the EV Drive and EV Brake modes. The lowest prediction performance is observed in the EV+Mechanical Brake and Mechanical Brake modes. The primary factor influencing the prediction accuracy is whether the engine is actively involved in vehicle propulsion. When the engine is engaged, its instantaneous fuel consumption exhibits a strong correlation with the driver’s accelerator pedal position. After the model has been trained on such strong correlations, it tends to maintain this relationship even when the vehicle switches to EV mode, where the engine should ideally consume no fuel. This results in a lag in the predicted fuel consumption, which fails to drop to zero as quickly as observed in real-world measurements.

Furthermore, the degree to which the motor contributes to braking also affects the model’s predictive performance. In regenerative braking scenarios, electric power output is strongly correlated with brake pedal position. However, when the braking mode gradually transitions to one dominated by mechanical braking, the model struggles to rapidly reduce the predicted electric power to zero. Additionally, due to the lack of an effective representation of the relationship between increasing mechanical brake pressure and enhanced deceleration, the predicted braking deceleration tends to be lower than the measured values, leading to a partial loss of dynamic response information.

To thoroughly investigate the impact of mode classification accuracy on downstream energy prediction performance, this study designs and conducts three comparative experiments centered on the ePower prediction task. The objective is to quantitatively assess how the accuracy of the critical mode information feature influences the final prediction results. The experimental setups are as follows: (1) **0 Mode**: No mode information is introduced during either the training or prediction phases, forcing the model to rely solely on raw driving behavior signals for learning and inference. (2) **10% error-Mode**: Based on the true mode labels, 10% of the data points are randomly selected and their mode labels are replaced with incorrect classes, simulating a real-world scenario where the mode recognition system has a 10% misclassification rate. (3) **True-Mode**: The model is trained and predicted using perfectly accurate mode labels, representing the ideal scenario of maximum mode recognition accuracy.

**Fig 13:**
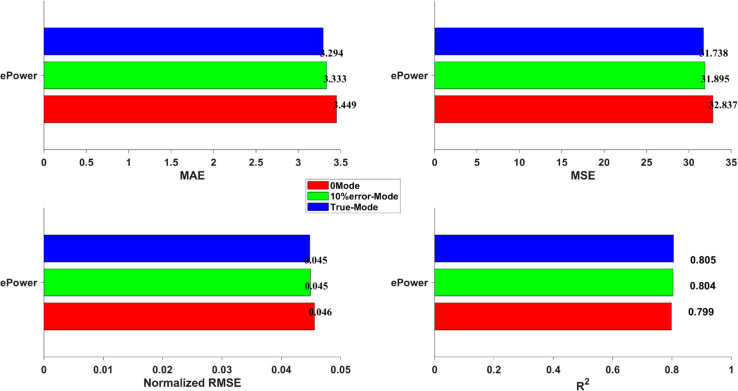
Comparison of PHEV ePower prediction under different mode label conditions.

As illustrated in Fig 13, the results of this comparative experiment clearly reveal a positive correlation between the accuracy of mode information and prediction performance. As the mode information evolves from 0 Mode to containing a 10% error rate, and finally to being completely accurate (True-Mode), the model demonstrates a consistent and significant performance improvement across four core evaluation metrics: MAE, MSE, Normalized RMSE, and *R*^2^. Specifically, taking MAE as an example, when transitioning from no mode input (0 Mode) to input with 10% erroneous mode labels (10% error-Mode), the MAE decreases from 3.4493 to 3.3295, an improvement of approximately 0.11. Furthermore, when advancing from 10% erroneous mode labels to perfectly accurate mode labels (True-Mode), the MAE decreases again to 3.2941, an additional improvement of approximately 0.03. This indicates that even a 10% misclassification rate in mode identification leads to an increase in prediction error, while providing more precise mode information can effectively reduce prediction bias.

In summary, the experimental results not only comprehensively validate the effectiveness of the DLLT’s dual-layer architecture but also highlight its significant superiority in capturing the complex mode operational characteristics of PHEVs. As the accuracy of mode identification improves, the model consistently demonstrates substantial performance gains across multiple evaluation metrics, including MAE, MSE, and *R*^2^. This further confirms that high-precision mode classification is a critical factor in achieving accurate energy consumption prediction. The analysis also reveals the decisive impact of mode information on downstream prediction tasks, providing important theoretical support and practical insights for the development of high-accuracy, interpretable intelligent energy prediction models.

## 5 Discussion

This paper presents a novel Dual-Layer LSTM-Transformer model for real-time prediction of energy consumption and driving dynamics in PHEVs. Experimental results demonstrate that the proposed model achieves superior performance in both vehicle mode identification and energy consumption prediction, significantly outperforming established baseline models such as LSTM, GRU, CBA, and standard Transformer architectures. The high predictive accuracy of DLLT offers strong potential for advancing intelligent energy management systems and enabling data-driven analysis of eco-driving behaviors in PHEVs.

To provide a comprehensive evaluation of the model’s value, limitations, and future prospects, a SWOT (Strengths, Weaknesses, Opportunities, and Threats) analysis is conducted.

1. **Strengths:** The primary strength of the DLLT model lies in its innovative hierarchical architecture. The first layer, based on a LSTM network, effectively captures the temporal dependencies inherent in driver behavior, achieving a high vehicle mode classification accuracy of 93.23%. The second layer employs a Transformer network that integrates the mode classification results as contextual information, leveraging a self-attention mechanism to fuse driver inputs (e.g., pedal operations) with vehicle state variables (e.g., speed, State of Charge). This enables highly accurate multi-output regression, achieving *R*^2^ scores of 0.99 for fuel consumption, 0.81 for electric power, and 0.86 for vehicle acceleration. This dual-layer design effectively addresses the complexity arising from the multi-mode operation of PHEVs, where energy sources dynamically switch between electric, hybrid, and internal combustion engine modes.
2. **Weaknesses:** Overall, the model performs well, but it is less accurate when it comes to braking and changing between driving modes. This is especially true when switching from engine-driven to pure electric drive, and from regenerative braking to pure mechanical braking, as illustrated in Figs 11 and 12. A noticeable lag appears in the DLLT’s predictions of fuel and electric power consumption. During transitions, the model fails to quickly reduce these values to zero, leading to delayed or inaccurate estimates. This limitation stems from the model’s learned strong correlation between the accelerator pedal position and engine fuel consumption. When the vehicle switches from engine-driven to pure electric mode, the model’s prediction exhibits inertia, resulting in a delayed drop in predicted fuel consumption. Similarly, during the transition from regenerative braking to pure mechanical braking, the model struggles to quickly adjust the predicted electric power to zero, indicating a challenge in capturing abrupt state changes and the complex dynamics inherent in mode switching.
3. **Opportunities:** The high accuracy and real-time capability of the DLLT model open up significant application opportunities. First, it can be integrated into in-vehicle systems as a real-time eco-driving assistant, providing drivers with immediate feedback on their energy consumption to promote more efficient driving habits. Second, the model can serve as a core component of a Predictive Energy Management System (P-EMS), where future energy demand is forecasted using navigation and traffic data, allowing the vehicle to pre-emptively optimize engine starts and battery charging/discharging for maximum efficiency. Furthermore, the DLLT framework provides a robust foundation for developing energy-efficient control strategies in connected and autonomous vehicles.
4. **Threats:** The model’s performance depends on the quality and representativeness of the training data. Further validation is required to confirm its ability to perform in real-world, uncontrolled environments such as those involving varying road grades, extreme temperatures or diverse driver populations. Additionally, the rapid evolution of deep learning architectures may soon yield more efficient or accurate models that could surpass DLLT. Finally, deploying such a computationally intensive model on the resource-constrained embedded systems of production vehicles presents significant challenges in terms of computational efficiency and real-time inference latency.

In conclusion, the DLLT model demonstrates significant advantages in the domain of real-time PHEV energy prediction, with substantial potential for practical applications. Future work will focus on mitigating the identified prediction lag during mode transitions and rigorously evaluating the model’s robustness across a wider range of real-world driving conditions to facilitate its deployment in actual vehicle systems.

## 6 Conclusion

This paper proposes DLLT, a novel dual-layer LSTM-Transformer model for real-time prediction of eco driving behavior in PHEVs. To address the complex multi-mode nature of PHEV operations, DLLT adopts a hierarchical architecture. The first layer uses an LSTM network to cluster vehicle operating modes over time, while the second layer employs a Transformer-based model to predict key outputs such as battery power, engine fuel consumption and vehicle acceleration by integrating both raw driver inputs and the predicted mode information. This integrated mode-aware design enables DLLT to effectively capture the nonlinear dynamics of multi-source energy flow, resulting in accurate identification of operational states and precise real-time predictions of energy consumption and vehicle dynamics. Evaluated against state-of-the-art deep learning models, DLLT demonstrates superior performance with a mode classification accuracy of 93.23 percent and *R*^2^ values of 0.9945 for fuel consumption, 0.8053 for electric power and 0.8565 for acceleration. These results highlight DLLT’s robust predictive capability and its potential for advancing eco-driving systems, intelligent energy management, and future autonomous vehicle control strategies. In future work, we will deploy and test DLLT on a wider range of PHEV models under diverse real-world driving conditions to further enhance its generalizability and practical applicability across the broader PHEV ecosystem.
